# Diplopia in Cases With Type 1 Duane Retraction Syndrome

**DOI:** 10.7759/cureus.15769

**Published:** 2021-06-20

**Authors:** Ramazan Birgul, Vuslat Gürlü

**Affiliations:** 1 Ophthalmology, Ministry of Health Kirikhan State Hospital, Hatay, TUR; 2 Ophthalmology, Trakya University, Edirne, TUR

**Keywords:** diplopia, stereopsis, amblyopia, duane retraction syndrome, fusion

## Abstract

Objective

In this study, we aimed to investigate the prevalence of diplopia in cases with type 1 Duane retraction syndrome (DRS).

Materials and methods

This study was a retrospective review of cases involving patients presenting diagnosed with DRS over a period of 24 years. Among these cases, 28 had type 1 DRS and fulfilled the inclusion criteria. The cases were evaluated in terms of age, gender, affected eye, concomitant ocular motility disorders, presence of amblyopia, manifest shift, abnormal head position (AHP), fusion, and stereopsis.

Results

Sixteen of the patients (57.1%) in the study were female, and 12 (42.8%) were male; the mean age of the patients was 18.9 years (range: 7-67 years). The right eye was affected in six of the cases (21.4%), and the left eye in 22 (78.6%) of the cases. On examination, diplopia was not observed in 21 (75%) cases, but it was detected in seven (25%). AHP was present in five of the seven cases with diplopia and not present in two, and all seven of the diplopic cases had fusion, while three had stereopsis. The level of stereopsis in all diplopic cases was 400 sn/ark. When the clinical findings of patients with diplopia and those without diplopia were compared, a statistically significant difference was observed only in terms of AHP.

Conclusions

Although diplopia is not one of the clinical features of DRS, it must be noted that in cases with type 1 DRS, diplopia may occur in directions in which the movement of the eyeball is limited. In the presence of this finding, which might mimic sixth nerve palsy, patient history must be diligently taken, other clinical findings of DRS must be thoroughly examined, and an MRI should be performed when necessary for an easier diagnosis.

## Introduction

Duane retraction syndrome (DRS) is a congenital syndrome characterized by globe retraction and the up or down shift of the affected eye at adduction, which may be accompanied by abduction and/or adduction limitations [1]. DRS is a congenital ocular motility disorder that usually occurs sporadically and is seen in 1-5% of all patients with strabismus. As per MRI findings and autopsy exams, the common causes of this condition are as follows: absence of the abducens nerve or its nucleus, and innervation of the lateral rectus by an aberrant branch of the oculomotor nerve [2-6]. In cases with DRS, surgical options are explored if abnormal head position (AHP) or overt horizontal shift is present [7-9].

AHP commonly occurs to establish fusion and repair the binocular dysfunction that may be caused by vertical deviations. The position of the head usually leans toward the weak muscle. This way, diplopia can be prevented while binocularity is preserved [2,4,10,11,12]. This is the reason why the presenting symptoms of the cases usually do not include diplopia and diplopia is not observed among the classical clinical findings of DRS; however, studies that examined the binocular statuses of DRS patients have reported cases with diplopia [13,14].

The aim of this study was to show that diplopia, which is included in the differential diagnosis of DRS and paralysis of the sixth nerve and is usually interpreted as part of the paralysis of the sixth nerve, may occasionally occur in patients with DRS and to investigate the prevalence of diplopia in cases with type 1 DRS, as well as its contributing factors.

## Materials and methods

This study was a retrospective review of diplopia cases. The principles of the Declaration of Helsinki for the use of human subjects were adhered to in this study. The study was approved by the Ethics Committee of the Trakya University for the purpose of clinical research with the title Diplopia in Cases With Type 1 Duane Retraction Syndrome, on 15/06/2016 with the protocol number TUTF-BAEK 2016/153.

The records of patients who were diagnosed with DRS and followed up between March 1990 and April 2014 in our Unit of Strabismus were retrospectively examined. Among these cases, 28 patients with type 1 DRS who had regular file records and who were old enough to define diplopia were included in the study.

The cases were evaluated in terms of age, gender, affected eye, concomitant ocular motility disorders, presence of amblyopia, manifest shift, AHP, fusion, stereopsis levels, and a diplopia test performed with objects. Care was taken to ensure that none of the patients had any history of birth traumas, head traumas, or neurological diseases. Moreover, none of the patients had systemic anomalies that may be seen in DRS, such as perceptive deafness, facial asymmetry, or cleft palate.

The age, gender, affected eye, concomitant ocular motility disorders, the existence of amblyopia, manifest shift, AHP, fusion, stereopsis levels, and the results of the diplopia test done with objects were obtained from the records of the cases.

The Huber classification was used for defining type 1 DRS. In this classification, DRS is divided into three types according to clinical findings and electromyographic test results:

• Type 1: limited abduction or normal or mildly limited adduction.

• Type 2: limited adduction and normal or mildly limited abduction.

• Type 3: overt limitation in both abduction and adduction.

Globe retraction and narrowing in the palpebral fissure are also observed in all three types. The cases may also be accompanied by an up or down shift of the affected eye at adduction.

The best-corrected visual acuities as determined by objective and subjective refractive methods, as well as the Snellen chart, were obtained from the file records. A best-corrected visual acuity of less than 8/10 was considered as "amblyopia". Cover tests were used for the determination of orthotropia, and in cases without orthotropia, the shift amount determined by prism cover tests, the existence of AHP, Bagolini and Worth 4 dot test results, and stereopsis levels determined by the Titmus test were used. If AHP was found, departments of pediatric surgery, ear, nose, and throat (ENT), and orthopedics were consulted to determine the etiology of AHP. An organic cause of AHP was not found. In the examination of the eyeball, areas with "looking limitations" in the primary and eight peripheral-looking positions were determined. The existence of diplopia was also determined using objects, and the quadrants with diplopia were noted. The cycloplegic exam was done 45 minutes after 1% cyclopentolate was dropped twice with five-minute intervals. After the determination of cycloplegic refraction, an ophthalmoscopy was performed.

The data were entered into the SPSS Statistics software (IBM, Armonk, NY), and chi-square tests were used for statistical analyses. A p-value <0.05 was considered statistically significant.

## Results

In our cohort, 16 of the patients (57.1%) were female, and 12 (42.8%) were male; their mean age was 18.9 years (range: 7-67 years). The right eye was affected in six of the cases (21.4%), and the left eye in 22 (78.6%) of the cases. Among the 22 orthophoric cases, 14 were orthophoric at the primary position, and eight were orthophoric with AHP. Among the five cases with esotropia, it was observed at the primary position in two cases, and it occurred along with AHP in three cases. Exotropia was also present at the primary position in one case, and this patient did not have AHP. Of note, 24 cases had a perfect vision (1.0). Amblyopia was strabismic in all four cases with amblyopia (14.2%). Varying degrees of AHP was present in 11 cases (39.2%). Stereopsis was present in eight cases, and fusion was present in 24 cases.

Diplopia was detected in seven of the cases, and the left eye was affected in all of them. In four of the six diplopic cases with orthophoria, orthophoria was observed with AHP in four and at the primary position in two. One case had esotropia with AHP. Diplopia was observed in three quadrants, which were the upper left, left, and lower left-looking positions in six of the cases. In one patient with esotropia, diplopia was present at the primary position as well as the looking-up position along with the left-looking position.

AHP was present in five of the seven cases with diplopia and not present in two, and all seven of the diplopic cases had fusion, while three had stereopsis. The level of stereopsis in all diplopic cases was 400 sn/ark.

Upon comparing the clinical findings of cases with and without defined diplopia, no significant difference was found in terms of age, gender, affected eye, visual acuity, type of shift, fusion, or stereopsis (c2 test; p=0.803, p=0.378, p=0.111, p=0.817, p=0.794, p=0.154, p=0.334, respectively); in fact, a significant difference was found only pertaining to AHP (p=0.044) (Tables 1, 2).

**Table 1:** Properties of cases with and without diplopia

Cases	Female	Male	Right eye	Left eye	Orthophoria	Esotropia	Exotropia	Amblyopia
With diplopia (n=7)	5	2	0	7	6	1	0	0
Without diplopia (n=21)	11	10	6	15	16	4	1	4

**Table 2:** Comparison of binocularity in patients with and without diplopia

	With diplopia (n=7)	Without diplopia (n=21)	P-value
Abnormal head position	5	6	0.044
Fusion	7	17	0.154
Stereopsis	3	5	0.334

## Discussion

Most studies on DRS in the literature have focused on clinical findings and surgical treatment results. In this study, the clinical properties of type 1 DRS, whether diplopia is observed in type 1 DRS, and the association between clinical features and diplopia were examined.

DRS is more frequently observed in females and in the left eye [15]. In our study, 57.1% of the patients were women, and 78.6% of these cases had the left eye affected, which is in line with findings in the literature. Of note, 40% of the cases with DRS were orthophoric. Esotropia has been reported to be the most common ocular deviation in many studies [16]. Esodeviations are most commonly observed in type 1 DRS, and the amount of shift is usually less than 30 prism dioptries. In our study, 78.6% of the cases had orthophoria, 17.8% had esotropia, and 3.6% had exotropia. The angle of the shift was less than 30 prism dioptries in all the cases. In addition, AHP was detected in 36.4% of the orthophoric cases and 60% of the esotropic cases, and AHP was not observed in the one case with exotropia.

The incidence rate of amblyopia has been reported to be between 3 and 25% in DRS [8,15,17,18]. The amblyopia rate in our study was determined to be 14.2%, which concurs with previous studies. Tomaç et al. [13] have reported the prevalence of AHP in DRS as 21% in one of their studies, whereas AHP was detected in 39.2% of the cases in our study.

The number of studies examining binocular visions in DRS cases is scarce in the literature. In their study that evaluated the binocularity of 29 cases with DRS, Tomaç et al. [13] reported that 83% of the cases had fusion, 24% of the cases were normal in terms of stereopsis, 52% had decreased stereopsis, and 24% had none. They detected diplopia in two of the 29 cases with the Worth 4 dot test and found a relationship between the level of stereopsis and the type of DRS, shift amount, and limitations in eyeball movement. They did not find a significant relationship between AHP and stereopsis. They concluded that DRS cases differ in terms of binocularity and clinical findings and that binocularity is better in type 1 DRS. In our study, we determined the level of fusion to be 86% and stereopsis to be 28.5%.

Kim et al. [14] have reported two cases with DRS whose presenting symptom was double vision that began in adulthood. One of these cases was diagnosed with Miller-Fisher syndrome and the other with bilateral abducens paralysis; however, a detailed evaluation of their ocular motilities and the presence of the typical findings of DRS led to the diagnosis of DRS, and the lack of the abducens nerve in MRI led these two cases to be confirmed with DRS. Kim et al. [14] have attributed the late and sudden onset of diplopia to the fibrosis of the lateral rectus muscle over time and the decrease of fusional divergence with age. None of the patients had double vision as a presenting symptom in our study, and the age range of those with diplopia was 14-52 years. The wide age range in diplopia onset shows that factors other than the fibrosis of the lateral rectus muscle and the decrease of fusional divergence with age play a role in its development.

Diplopia, which is not one among the classical clinical findings of DRS, had a prevalence of 25% in our study. The aim of detecting AHP in cases is to prevent diplopia by preserving the continuity of binocularity in at least one quadrant [2]. In addition, the fact that the p-value between diplopia and AHP was 0.044 shows that the relationship between the two is significant. All seven cases with diplopia had fusion, while only three had stereopsis. This result shows that AHP develops as compensation, and while fusion is preserved, stereopsis is not; and hence, diplopia is not prevented.

## Conclusions

Although diplopia is not one of the clinical properties of DRS, it must be noted that in cases with type 1 DRS, diplopia may occur in directions in which the movement of the eyeball is limited. If this finding is detected, which might mimic sixth nerve palsy, patient history must be carefully and thoroughly taken, the other clinical findings of DRS must be rigorously examined, and an MRI should be performed when necessary for an easier diagnosis.
